# Inducible Costimulator and Its Ligand Promote Proliferation and Migration of Tumor Cells in Cutaneous T-Cell Lymphoma

**DOI:** 10.3390/ijms27031408

**Published:** 2026-01-30

**Authors:** Kenta Oka, Takuya Miyagawa, Hiromichi Morita, Hiraku Suga, Tomomitsu Miyagaki, Sayaka Shibata, Hiroaki Kamijo, Yuka Mizuno, Teruyoshi Hisamoto, Issei Omori, Hikari Boki, Tomonori Oka, Naomi Takahashi-Shishido, Makoto Sugaya, Shinichi Sato

**Affiliations:** 1 Department of Dermatology, Graduate School of Medicine, The University of Tokyo, Hongo, Bunkyo-ku, Tokyo 113-8655, Japan; 2 Department of Dermatology, St. Marianna University School of Medicine, Sugao, Miyamae-ku, Kanagawa 216-8511, Japan; asahikari1979@gmail.com; 3 Department of Dermatology, International University of Health and Welfare, Kouzunomori, Chiba 286-8686, Japan

**Keywords:** cutaneous T-cell lymphoma, inducible co-stimulator, inducible co-stimulator ligand, mycosis fungoides, Sézary syndrome, CCR4

## Abstract

Inducible costimulator (ICOS) is a costimulatory immune checkpoint receptor expressed on activated T-cells, while the ICOS ligand (ICOSL) is expressed on antigen-presenting cells. The ICOS–ICOSL axis promotes the survival of memory and effector T-cells and induces several immune responses. In addition, the ICOS–ICOSL interaction induces cell proliferation, cell survival, and cytokine production. The roles of ICOS and ICOSL in cutaneous T-cell lymphoma (CTCL) are unclear. In this study, we examined the roles of ICOS and ICOSL in CTCL. The tumor cells co-expressed ICOS and ICOSL, and the upregulated expression of ICOS and ICOSL reflected disease severity. Anti-ICOS and anti-ICOSL neutralizing antibodies inhibited both the in vitro and in vivo proliferation of CTCL cell lines. The anti-ICOSL neutralizing antibodies induced apoptosis and suppressed CCR4 expression on tumor cells, inhibiting CCR4–CCL17-mediated migration. These results suggest that the ICOS–ICOSL axis plays an essential role in CTCL pathogenesis, and targeting the ICOS–ICOSL axis could be a viable strategy for treating CTCL.

## 1. Introduction

Mycosis fungoides (MF) and Sézary syndrome (SS) are common types of cutaneous T-cell lymphoma (CTCL) [1]. MF is the most classical type of CTCL, characterized by the malignant proliferation of neoplastic CD4^+^ T-cells with epidermotropism in the skin, showing a typical prolonged clinical course. SS is characterized by the triad of erythroderma, lymphadenopathy, and leukemic involvement, which usually show rapid progression. Although various treatments for patients with advanced MF and SS are currently available, the effects of these therapies are limited, and the only option for improving a prognosis of advanced MF and SS is stem cell transplantation [2,3,4]. Therefore, novel curative therapies are desired.

In CTCL, interactions between tumor cells contribute to the proliferation of malignant growth through paracrine or autocrine mechanisms. Cytokines secreted from tumor cells, such as interleukin (IL)-13 and IL-15, contribute significantly to tumor cell progression [5,6,7]. Surface molecules, such as CD40 and CD28, promote tumor growth through various signaling pathways [8,9,10].

Inducible costimulator (ICOS), a member of the CD28 coreceptor family, is a costimulatory immune checkpoint receptor [11]. Unlike CD28, ICOS is not expressed on naïve T-cells but on activated T-cells. ICOS plays various roles in immune responses, and cytokines, such as IL-4 and IL-13, are released by ICOS, leading to the proliferation and differentiation of T-cells [11,12]. Moreover, ICOS has been shown to promote antitumor immune responses. Based on this effect, anti-ICOS agonist antibodies have shown antitumor effects in treating malignant tumors [13,14]. In contrast, several roles played by ICOS in malignancy have been reported. In malignant melanoma, ICOS^+^ T regulatory (Treg) cells express IL-10, which suppresses antitumor immunity [15]. Furthermore, tumor cells themselves express ICOS in several types of malignancies [16,17,18]. Marafioti T. et al. revealed that ICOS expression was confined to angioimmunoblastic T-cell lymphoma, comprising peripheral T-cell lymphomas of the follicular variant [17]. In addition, they also detected that ICOS worked as a useful marker for the diagnosis of AITL and PTCL-NOS [17]. In primary cutaneous CD4^+^ small/medium pleomorphic T-cell lymphoma, though the expression was less prevalent than PD-1, the tumor cells expressed ICOS [18].

ICOS ligand (ICOSL) is expressed on antigen-presenting cells, including B cells and dendritic cells [19]. ICOSL provides costimulatory signals for T-cells through ICOS [19,20]. ICOSL-expressing immature antigen-presenting cells induce the antigen-mediated activation of memory T-cells [21,22]. In addition, stimuli from ICOSL are transmitted to antigen-presenting cells, affecting cell migration and cytokine production [23,24]. Notably, ICOSL is aberrantly expressed in several malignancies, providing a favorable environment for tumor proliferation [25,26]. Tumor cells in malignant melanoma aberrantly express ICOSL, which promotes Treg activation [25]. Aberrantly expressed ICOSL promotes the sustained CD25 and Foxp3 expression of Tregs and provides costimulation through ICOS, IL-10-secreting Tregs with T-cell suppressive properties [25]. In acute myeloid leukemia, tumor cells express the ICOS ligand, promoting the expansion of ICOS^+^ Tregs in the tumor environment. These ICOS^+^ Tregs further promote the proliferation of tumor cells through the secretion of IL-10 [26].

Previous studies have revealed that ICOS is expressed in CTCL [27,28]; however, the roles played by the ICOS–ICOSL axis in CTCL are unclear. With regard to costimulatory molecules, we have demonstrated that in CTCL, the CD137–CD137 ligand (CD137L) and the OX40–OX40 ligand (OX40L) play critical roles in tumor cell proliferation [29,30]. The aim of this study was to examine the roles of ICOS–ICOSL in CTCL.

## 2. Results

### 2.1. Expression of ICOS and ICOSL in the Lesional Skin and Sera of CTCL Patients

**Figure 1:**
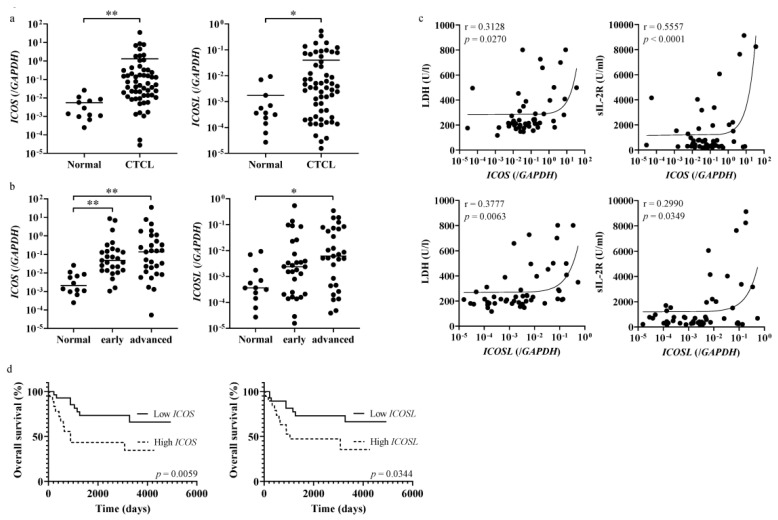
Expression of ICOS and ICOSL in patients with cutaneous T-cell lymphoma and healthy controls. (**a**,**b**) The expression of ICOS and ICOSL mRNA in the lesional skin of patients with cutaneous T-cell lymphoma (CTCL) (total *n* = 58; early *n* = 29, advanced *n* = 29) and the normal skin of healthy controls (*n* = 12). The statistical analysis was performed using the Mann–Whitney *U*-test. (**c**) The correlation between serum lactate dehydrogenase (LDH) or soluble interleukin-2 receptor (sIL-2R) levels and ICOS or ICOSL mRNA expression. Each curve represents the correlation line. The correlation coefficients were determined using the Spearman’s rank correlation test. (**d**) The Kaplan–Meier analysis of the overall survival between the patients with higher and lower ICOS or ICOSL expression. The differences in overall survival were assessed using the log-rank test. The optical cut-off points were determined by a receiver operating characteristic analysis. * *p* < 0.05, ** *p* < 0.01.

To examine the ICOS–ICOSL interaction in CTCL, we measured the levels of ICOS and ICOSL mRNA in CTCL lesional skin. Both ICOS and ICOSL expression in CTCL lesional skin were significantly elevated compared to normal controls (Figure 1a). Based on the clinical stage, ICOS and ICOSL mRNA levels were significantly elevated in patients with advanced stages of CTCL compared with normal controls (Figure 1b). ICOS and ICOSL mRNA expression was positively correlated with serum lactate dehydrogenase (LDH) or soluble IL-2 receptor (sIL-2R) levels (Figure 1c). We analyzed the association between ICOS–ICOSL mRNA expression and overall survival using the Kaplan–Meier method. Patients with high expression of ICOS mRNA had significantly lower survival rates than those with lower ICOS expression (Figure 1d). Consistently, patients with high ICOSL mRNA expression had lower survival rates (Figure 1d). To evaluate soluble forms of ICOS and ICOSL in CTCL, we measured the serum levels of each; however, these were below detection levels in both the CTCL patients and the healthy controls.

### 2.2. ICOS and ICOSL Expression on the Malignant T-Cells of CTCL Patients

**Figure 2:**
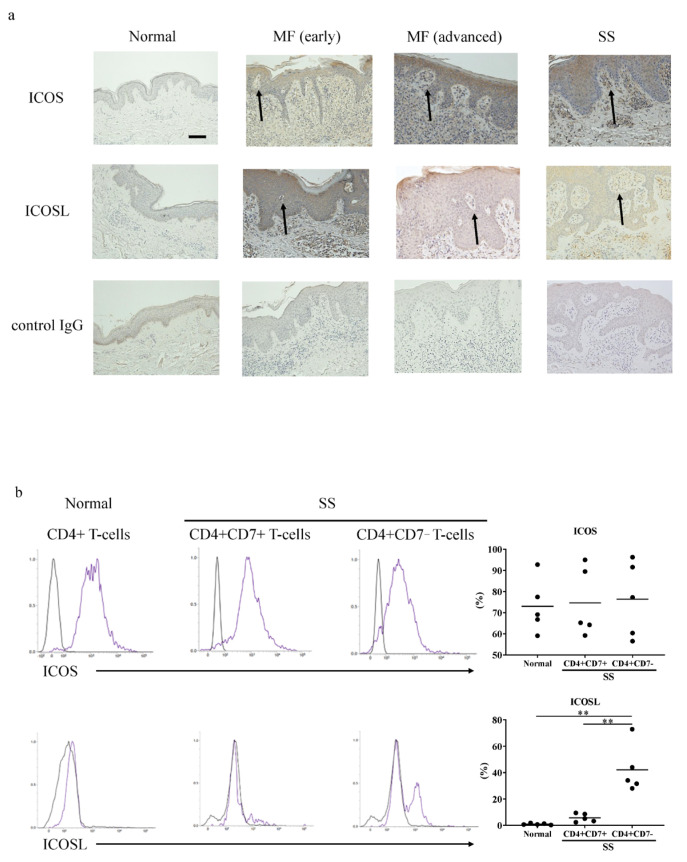
Immunohistochemical and flowcytometric analysis of ICOS and ICOSL in patients with cutaneous T-cell lymphoma and normal controls. (**a**) The expression of ICOS, ICOSL, and the control IgG was evaluated by immunohistochemistry in skin sections from patients with mycosis fungoides (MF) (total *n* = 26; early *n* = 11, advanced *n* = 15), Sézary syndrome (SS) (*n* = 5), and healthy controls (*n* = 9). The arrows represent the well-stained areas. Representative results are shown. The scale bars represent 50 μm. (**b**) ICOS- and ICOSL-positive cell ratios in peripheral blood T-cells between the patients with SS (*n* = 5) and the healthy controls (*n* = 5) were compared by flow cytometry. Black curves represent isotype. The statistical analysis was performed using the Mann–Whitney *U*-test. ** *p* < 0.01.

Because the expression of ICOS and ICOSL mRNA in lesional skin was increased in CTCL, we next assessed ICOS and ICOSL expression in the skin samples by immunohistochemistry. The immunohistochemical staining revealed that in the CTCL skin, the tumor cells strongly expressed ICOS and ICOSL, including the infiltrating lymphocytes in the epidermis (Figure 2a and Figure S1). The immunohistochemical analysis of all cases in each group is summarized in Table 1. The expression intensity in the tumor cells was quantified by ImageJ version 1.53e image analysis software, and the expression levels were significantly elevated both in the early and advanced CTCL patients compared to the healthy controls (Figure S1).

We analyzed the expression of ICOS and ICOSL in the CTCL cells with flow cytometry. Peripheral blood CD4^+^CD7^−^ T-cells from the patients with SS, which are considered tumor cells [31], expressed both ICOS and ICOSL. In contrast, the normal CD4^+^ T-cells from healthy controls and CD4^+^CD7^+^ T-cells from patients with SS expressed only ICOS (Figure 2b). Thus, in CTCL, tumor cells co-express ICOS and ICOSL.

### 2.3. Expression and Function of ICOS and ICOSL in CTCL Cell Lines

**Figure 3:**
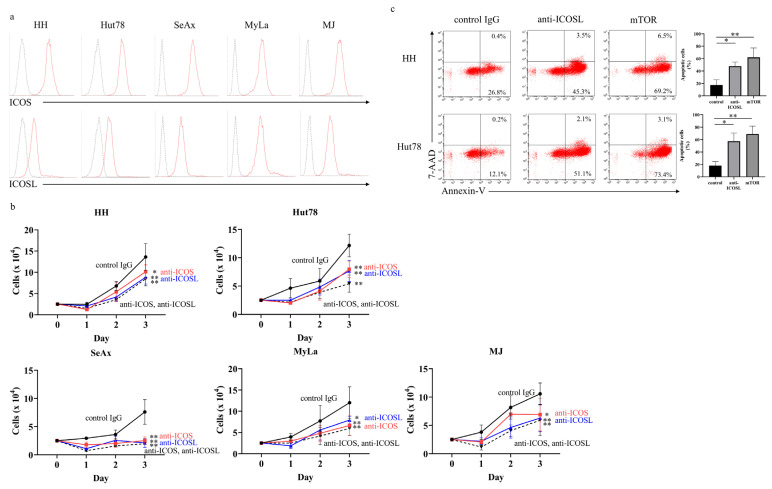
Involvement of ICOS and ICOSL in cutaneous T-cell lymphoma cell lines. (**a**) ICOS and ICOSL expression was evaluated by flow cytometry in human CTCL cell lines. (**b**) HH, Hut78, SeAx, MyLa, and MJ cells (2.5 × 10^4^ cells/well) were cultured with anti-ICOS (5 µg/mL) and/or anti-ICOSL (5 µg/mL) neutralizing antibodies. The statistical analysis was performed using the Mann–Whitney *U*-test. (**c**) HH and Hut78 cells (2.0 × 10^5^ cells/well) were cultured with anti-ICOSL (5 µg/mL) neutralizing antibody, everolimus, or the control IgG for 24 h. Apoptosis was evaluated with flow cytometry using Annexin V and 7-AAD staining. The statistical analysis was performed using the Mann–Whitney *U*-test. mTOR: everolimus. * *p* < 0.05, ** *p* < 0.01.

We first confirmed the expression of ICOS and ICOSL on the surfaces of the CTCL cell lines by flow cytometry. All five cell lines co-expressed ICOS and ICOSL on their surfaces (Figure 3a). Next, we blocked the ICOS–ICOSL axis with neutralizing antibodies to assess the role of the ICOS–ICOSL interaction in CTCL. The anti-ICOS or anti-ICOSL neutralizing antibody significantly suppressed the proliferation of each cell line, and both antibodies together had a similar effect (Figure 3b). We conducted the BrdU assay to examine the effects of anti-ICOS or anti-ICOSL neutralizing antibodies on the proliferation of cell lines. Each neutralizing antibody significantly suppressed the uptake of BrdU, more so than the normal controls (Figure S2). In addition, the anti-ICOSL neutralizing antibody induced apoptosis in the cell lines (Figure 3c).

### 2.4. Examination of Signaling Pathways

**Figure 4:**
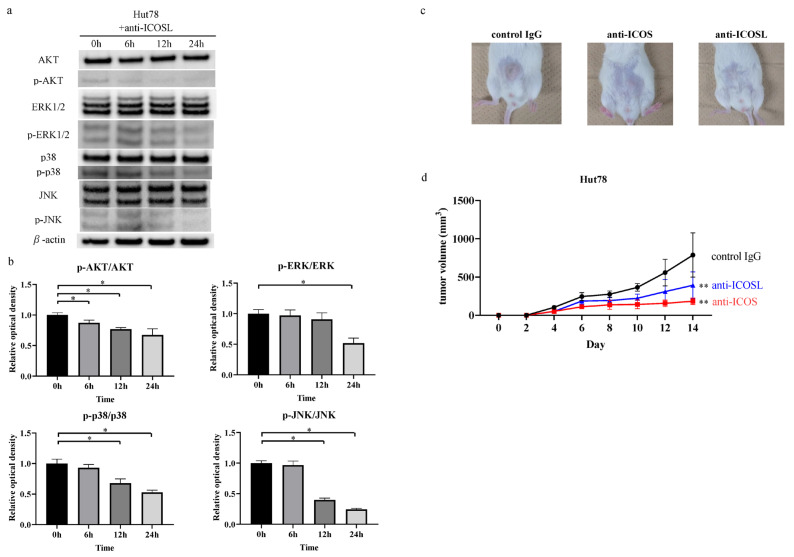
Western blotting analysis and in vivo effects. (**a**) Hut78 cells were cultured with anti-ICOSL neutralizing antibody (5 µg/mL) for 0, 6, 12, and 24 h for the phosphorylation of AKT, ERK1/2, p38 MAPK, and JNK. (**b**) Changes in the phosphorylation of the signal transduction molecules. The intensities of each signaling molecule were measured using ImageJ version 1.53e. The relative optical density of p-AKT/AKT, p-ERK/ERK, p-p38 MAPK/p38 MAPK, and p-JNK/JNK was calculated and normalized to the value of the “0 h” group. The statistical analysis was performed using the Mann–Whitney *U*-test. (**c**) Hut78 cells (5.0 × 10^6^ cells) in 100 µL of PBS were injected subcutaneously into the abdomen of NOD/SCID interleukin-2 receptor γ-chain-deficient (NSG) mice. Anti-ICOS, anti-ICOSL neutralizing antibody, or isotype IgG were injected on days 0, 4, 7, 11. Representative images are shown. (**d**) Tumor volume was evaluated using the following equation: V = π (L1 × L2)/6, where V = volume (mm^3^), L1 = the longest diameter (mm), and L2 = the shortest diameter (mm). The statistical analysis was performed using the Mann–Whitney *U*-test. * *p* < 0.05, ** *p* < 0.01.

Previous studies have indicated that downstream of the ICOS–ICOSL interaction lies PI3-kinase–AKT and MAP kinase signaling [32,33]. Consistently, the Western blot analyses revealed that the anti-ICOSL neutralizing antibody suppressed the phosphorylation of AKT, ERK1/2, p38 MAPK, and JNK in the Hut78 cells (Figure 4a,b). These results suggest that the anti-ICOSL neutralizing antibody suppressed the phosphorylation of intracellular signaling pathways. The relative optical density of p-AKT/AKT, p-ERK/ERK, p-p38 MAPK/p38 MAPK, and p-JNK/JNK was calculated and normalized to the value of the “0 h” group. Each ratio is shown in Table S1.

### 2.5. Examination of Tumor Growth In Vivo

We used a xenograft model to assess the in vivo function of the anti-ICOS and anti-ICOSL antibodies [34]. Treatment with the anti-ICOS and anti-ICOSL antibodies significantly decreased tumor growth in the Hut78 cells in the NSG mice in vivo (Figure 4c,d). Therefore, blocking the ICOS–ICOSL interactions suppressed the proliferation of tumor cells in CTCL both in vitro and in vivo.

### 2.6. Effect of ICOS–ICOSL on CCR4

To examine other potential effects of ICOS–ICOSL, we performed an RNA-Seq analysis using the total RNA extracted from the Hut78 cells co-cultured with the anti-ICOS or anti-ICOSL neutralizing antibody (Figure S3). Among the genes affected by the treatment, we focused on CCR4 since it plays important roles in the cell proliferation and migration of CTCL, and its monoclonal antibody, Mogamulizumab, is now used widely for the treatment of CTCL [35,36,37,38]. Therefore, we next examined the association between ICOS–ICOSL and CCR4.

**Figure 5:**
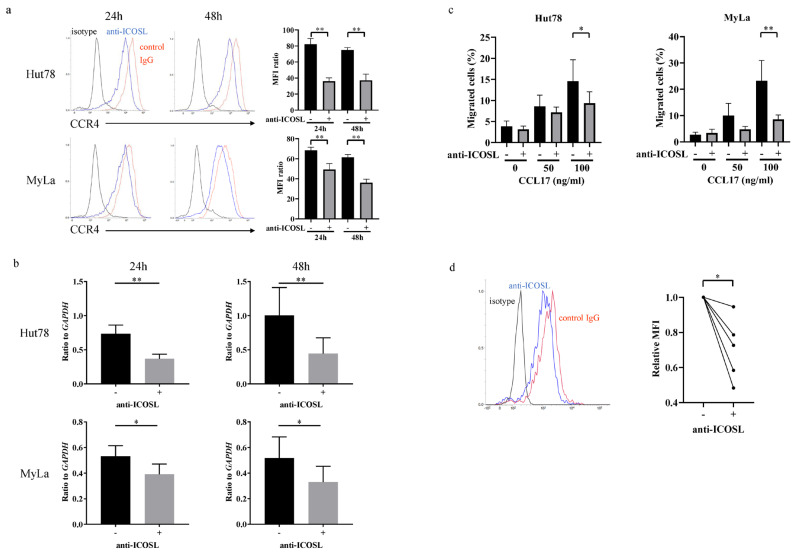
The effects of ICOSL on CCR4. Hut78 and MyLa cells were cultured with the anti-ICOSL neutralizing antibody (5 µg/mL) for 24 or 48 h. ICOSL expression was evaluated by flow cytometry (**a**) and quantitative RT-PCR (**b**). The mean fluorescence intensity (MFI) ratio was determined as the MFI of the target molecule/MFI of the isotype control. (**c**) Hut78 and MyLa cells were cultured with the anti-ICOSL neutralizing antibody (5 µg/mL) for 12 h, and their migration was assessed for 12 h at 37 °C with the human CCL17 protein (0, 50, 100 µg/mL). The percentage of migrated cells relative to input was evaluated. The statistical analysis was performed using the Mann–Whitney *U*-test. (**d**) Peripheral blood T-cells in patients with Sézary syndrome (SS) were cultured with the anti-ICOSL neutralizing antibody (5 µg/mL) for 24 h. CCR4 expression on the CD4^+^CD7^−^ T-cells was evaluated by flow cytometry. Relative MFI was determined as the ratio of the MFI of ICOSL- to the MFI of the isotype-treated cells. The Wilcoxon signed-rank test was used to examine the differences. Representative results are shown. * *p* < 0.05, ** *p* < 0.01.

The anti-ICOSL neutralizing antibodies significantly downregulated CCR4 surface protein and mRNA expression in the Hut78 and MyLa cells (Figure 5a,b). Treatment with the anti-ICOSL neutralizing antibodies significantly suppressed CCR4–CCL17-mediated migration (Figure 5c). The anti-ICOSL neutralizing antibody significantly downregulated CCR4 expression in malignant cells in the patients with SS (Figure 5d). Therefore, blocking ICOS–ICOSL suppressed CCR4 expression and CCR4–CCL17-mediated migration.

## 3. Discussion

We demonstrated that CTCL cells aberrantly express not only ICOS but also ICOSL. Consistently, ICOSL was ectopically expressed in other malignancies [25,26]. In malignant melanoma, tumor cells express ICOSL, activating Treg cells and inducing the secretion of IL-10, which suppresses tumor immunity [25]. In acute myeloid leukemia, tumor cells aberrantly express ICOSL, which activates Treg cells and creates a favorable environment for tumor proliferation [26]. These studies suggest that the aberrant expression of ICOSL promotes tumor cell proliferation and creates a favorable environment for malignant cells through cytokines such as IL-10 [39,40]. In CTCL, malignant cells adopt Treg phenotype, secreting IL-10 [41,42]. In addition, the elevated expression of IL-10 in CTCL has been reported [43,44]. The aberrant expression of ICOSL in CTCL might induce the activation of Treg cells and the secretion of IL-10, as mentioned above. In the current study, the mRNA expression of ICOS and ICOSL increased as the stage progressed and significantly correlated with serum LDH and sIL-2R, indicating the disease severity of CTCL. Furthermore, patients with higher expression of ICOS or ICOSL had significantly lower survival rates than those with low expression of ICOS or ICOSL. Thus, the higher expression of ICOS and ICOSL can contribute to the progression of CTCL.

We next found that the co-expression of ICOS and ICOSL on CTCL cells was involved in tumor proliferation, growth, and survival. Neutralizing ICOS or ICOSL antibodies significantly suppressed proliferation of the cell lines. To examine whether the neutralizing antibodies inhibited cell proliferation, we conducted the BrdU assay. We found that the uptake of BrdU was significantly suppressed in the anti-ICOS or anti-ICOSL neutralizing antibody-administered group. This result supported the effect of ICOS and ICOSL on the proliferation of the cell lines. We also conducted an apoptosis assay to determine whether anti-ICOS neutralizing antibodies induced apoptosis. We used everolimus as a positive control. We confirmed that the neutralizing antibody induced apoptosis in the CTCL cell lines. Though the difference was not statistically significant, the ratio of cell lines undergoing apoptosis was lower than it was in the everolimus-administered group. We speculated that one of the reasons was that the ICOS–ICOSL axis was reported to affect PI3-kinase–AKT, and mTOR is downstream of AKT. Since mTOR is located further downstream than ICOS, the tumor cells underwent apoptosis more rapidly. This difference in rapidity might ultimately have led to the disparity in the ratio; however, further investigation is needed to consider other mechanisms.

In addition to PI3-kinase–AKT, the ICOS–ICOSL axis has been reported to affect MAP kinase signaling downstream [32,33]. Therefore, we analyzed changes in the phosphorylation of these signaling molecules. The anti-ICOSL neutralizing antibody significantly suppressed the expression of phosphorylated AKT, ERK1/2, p38 MAPK, and JNK. Finally, we evaluated the effects of the anti-ICOS and anti-ICOSL neutralizing antibodies in vivo with immunodeficient mice models. The anti-ICOS or anti-ICOSL neutralizing antibodies significantly suppressed the tumor volume in the CTCL cells in an immunodeficient mice model when compared with the control group. These results suggest that the ICOS–ICOSL axis has an essential role to play in the pathogenesis of CTCL.

We performed an RNA-Seq analysis to examine whether the ICOS–ICOSL interaction affects CTCL through other mechanisms. The RNA-Seq analysis revealed that anti-ICOS and anti-ICOSL neutralizing antibodies downregulated CCR4 expression. CCR4 is a chemokine receptor that is highly expressed on tumor cells in patients with MF or SS [35,36]. Mogamulizumab, a humanized anti-CCR4 monoclonal antibody, has been used in clinical practice to treat relapsed or refractory CTCL [37,38]. Therefore, we examined the effects of ICOS and ICOSL on CCR4. Surprisingly, the anti-ICOSL neutralizing antibody significantly suppressed CCR4 expression and inhibited CCR4-mediated migration. In patients with atopic dermatitis, the stimulation of peripheral blood CD4^+^ T-cells by recombinant ICOSL increased CCR4 expression [45]. Although little is known about the association between ICOSL and CCR4, these results do not contradict this report. Further study is required to examine this association.

CD137–CD137L and OX40–OX40L, which, like ICOS–ICOSL, are co-stimulating factors, reportedly play a crucial role in CTCL progression [29,30]. Both CD137 and OX40 are members of the tumor necrosis factor receptor superfamily and are expressed on activated T- and NK cells [46,47]. We found that CD137–CD137L and OX40–OX40L were co-expressed on the CTCL cells. The CD137–CD137L and OX40–OX40L interaction promoted tumor cell survival and proliferation and might be a therapeutic target against CTCL [29,30]. In this study, we showed that the CTCL cells co-expressed ICOS and ICOSL, and the ICOS–ICOSL interaction played an important role in CTCL progression. Thus, targeting ICOS and ICOSL might be a viable strategy for treating CTCL.

Because signaling from ICOS exerts an anti-tumor effect [13], anti-ICOS agonist antibodies may be a viable treatment for malignant tumors. Consistently, clinical trials with anti-ICOS agonist antibodies, such as GSK3359609 and JTX2011, have been performed against carcinomas such as echinocellular carcinoma of the head, non-small cell lung cancer, and severe pleural neutrophilic tumors [48,49]. However, the benefits from these immunotherapies have varied greatly across patients, and the therapeutic effect is often limited. One of the reasons for this is that the effectiveness of immunotherapy treatment is dependent on the immune status of the patient. To improve its effect, studies have been using immunotherapy in combination with other immunotherapies or chemotherapy to improve the therapeutic effect. We showed that unlike other malignancies, CTCL cells co-expressed ICOS and ICOSL, and the ICOS–ICOSL interaction is crucial for the proliferation of tumor cells. This interaction may be involved in tumor progression through mechanisms other than the tumor immune response. A treatment strategy which enhances the antitumor effects of the ICOS agonist may activate tumor cell growth in CTCL. Thus, for malignant tumors that co-express ICOS and ICOSL, therapies targeting the ICOS–ICOSL interaction may be more effective than immunotherapy.

Our study had several limitations. As noted in the introduction, interactions between tumor cells in CTCL contribute to the proliferation of malignant growth through paracrine or autocrine mechanisms, and the cytokines secreted from tumor cells, such as interleukin IL-13, contribute to tumor cell progression [5,6,7]. The ICOS–ICOSL axis affects autocrine/paracrine signaling through cytokines such as IL-4 and IL-13, leading to the proliferation of T-cells [11,12]. We did not show how the ICOS–ICOSL axis affects these cytokines in this study. Further study is required to elucidate how the ICOS–ICOSL axis directly affects tumor proliferation. In addition, for the in vivo experiment, we used NSG mice, which do not have functional immune systems. Because normal immune cell functions, such as those of normal B- and T-cells, are absent in this mouse model, further studies are needed to determine how blocking the ICOS–ICOSL interaction affects the immune response to tumor cells. Also, the effects of inhibiting this interaction on normal skin were not determined. While the use of mice reflecting the pathogenesis of CTCL may be desirable, no such mouse model has been established to date.

In conclusion, we showed that in CTCL, tumor cells co-express ICOS and ICOSL. Since the ICOS–ICOSL interaction contributes to tumor cell proliferation, the inhibition of this interaction could prove a viable therapeutic strategy for treating CTCL.

## 4. Materials and Methods

### 4.1. Patients

We diagnosed MF and SS based on the criteria of the International Society for Cutaneous Lymphomas and the cutaneous task force of the European Organization of Research and Treatment of Cancer [1]. Lesional skin samples were obtained from 52 patients with MF, 6 patients with SS, and 12 healthy controls for the RT-PCR. The patients with CTCL were sub-grouped into early (IA, IB, and IIA; *n* = 29) and advanced (IIB, III, and IV; *n* = 29) stages. Lesional skin samples were obtained from 26 patients with MF, 5 patients with SS, and 9 healthy controls for the immunohistochemistry. The patients with MF were sub-grouped into early (IA, IB, and IIA; *n* = 11) and advanced (IIB, III, and IV; *n* = 15) stages. Serum samples were collected from 40 patients with MF, 10 patients with SS, and 15 healthy controls. Peripheral blood mononuclear cells (PBMCs) were obtained from 5 patients with SS and 5 healthy controls. The healthy controls had no history of allergic disease or CTCL. All samples were collected during daily clinical practice. The medical ethical committee of the University of Tokyo approved all studies. This study was conducted according to the principles of the Declaration of Helsinki (approval number: 0695-(17)). Written informed consent was obtained from the patients.

### 4.2. Cell Lines

HH cells (an aggressive CTCL cell line), Hut78 and SeAx cells (SS cell lines), and MyLa and MJ cells (MF cell lines) were kind gifts from Dr. Kazuyasu Fujii (Department of Dermatology, Kagoshima University, Kagoshima, Japan). The cell lines were cultured in RPMI-1640 with 10% fetal bovine serum and supplements (penicillin G sodium, streptomycin sulfate, and amphotericin B).

### 4.3. Quantitative RT-PCR

RNA was extracted from human skin samples using an RNeasy Fibrous Tissue Mini Kit (Qiagen, Valencia, CA, USA). Total RNA was extracted from the CTCL cell lines with Direct-zol RNA Microprep kits (Zymo Research, Irvine, CA, USA). Quantitative RT-PCR was performed based on the SYBR Green assay. mRNA levels were normalized to those of the Glyceraldehyde-3-phosphate dehydrogenase (*GAPDH*) gene. Relative changes in gene expression were determined by the 2^−∆∆Ct^ method. The primers used were as follows: –*ICOS* forward: 5′-GTG CTC ACT GGG AGT GGA AT-3′ and reverse: 5′-GTC AAC TGG GTT CAG CAA T-3′; *ICOSL* forward: 5′-CGT GTA CTG GAT CAA TAA GAC GG-3′ and reverse: 5′-TGA GCT CCG GTC AAA CGT GGC C-3′; and *CCR4* forward: 5′-TAA TAT TGC AAG GCA AAG ACT ATT CC-3′ and reverse: 5′-GCG ATT TAC TCC ATC AGC CAG TA-3′.

### 4.4. Enzyme-Linked Immunosorbent Assay

ICOS and ICOSL in sera were quantified using a Human ICOS (CD278) ELISA Kit (Invitrogen, Carlsbad, CA, USA) and a Human B7-H2 ELISA Kit (RayBiotech, Norcross, GA, USA). Optical densities were measured at 450 nm using a Bio-Rad Model 550 microplate reader (Bio-Rad Laboratories, Hercules, CA, USA). Concentrations were calculated from the standard curve generated by a curve-fitting program.

### 4.5. Immunohistochemistry

Briefly, 5 µm-thick tissue sections from formaldehyde-fixed and paraffin-embedded samples were dewaxed and rehydrated. After rehydration, the sections were autoclaved in 10 mM of sodium citrate buffer for antigen retrieval and stained with rabbit ICOS antibody (Biorbyt, Cambridge, UK), rabbit anti-ICOS Ligand/ICOSL antibody (Abcam, Cambridge, MA, USA), and rabbit IgG, polyclonal (Abcam) as the isotype control, followed by ABC staining (Vector Lab, Burlingame, CA, USA). Diaminobenzidine was used as a chromogenic substrate, according to the manufacturer’s instructions. The intensity of staining in the epidermis was visually quantified as follows: -, no staining; +, slight staining; ++, moderate staining; and +++, strong staining. The intensity was also quantified by ImageJ version 1.53e image analysis software (National Institutes of Health, Bethesda, MD, USA) and compared between the CTCL skin samples and the healthy controls.

### 4.6. Flow Cytometry

We conducted a flow cytometric assay with the CTCL cell lines and PBMCs from the patients with SS and the healthy controls. The antibodies used were as follows: PE anti-human/mouse/rat CD278 (ICOS) antibody (clone C398.4A; BioLegend, San Diego, CA, USA), PE/Cyanine 7 anti-human CD275 (B7-H2, ICOSL) antibody (clone 2D3; BioLegend), PE anti-human CD194 (CCR4) antibody (clone L291H4; BioLegend), anti-human CD4-APC (clone 13B8.2; Beckman Coulter, Miami, FL, USA), and anti-human CD7-FITC (clone 8H8.1; Beckman Coulter).

### 4.7. Proliferation Analysis

HH, Hut78, MJ, MyLa, and SeAx cells were co-cultured with the anti-ICOS or anti-ICOSL neutralizing antibody. The cells were collected after 24, 48, and 72 h, respectively, and the number of viable cells was counted after staining with trypan blue. Anti-human ICOS (5 μg/mL; Novus Biologicals, Centennial, CO, USA) and anti-human ICOSL (5 μg/mL; R&D Systems, Minneapolis, MN, USA) were used as the neutralizing antibodies.

### 4.8. BrdU Assay

We used Cell Proliferation ELISA, BrdU (colorimetric) (Sigma-Aldrich, St. Louis, MO, USA) for the BrdU assay. HH and Hut78 cells were co-cultured with the anti-ICOS or anti-ICOSL neutralizing antibody. After 24 h of incubation, we added the BrdU solution and incubated for another 2 h. Optical densities were measured at 405 nm and 650 nm using Multiskan FC (Thermo Fisher Scientific, Cleveland, OH, USA), and we determined the differences in the optical densities as the absorbance.

### 4.9. Apoptosis Assay

HH and Hut78 cells were co-cultured with the anti-ICOSL neutralizing antibody and the control IgG. Everolimus was used as the positive control. The cells were collected after 24 h, respectively. The number of apoptotic cells was evaluated by a flow cytometric assay with Annexin V (conjugated FITC) and 7-amino-actinomycin D (7-AAD) (BioLegend) as the antibodies.

### 4.10. Western Blotting

Hut78 cells were co-cultured with the anti-ICOSL antibody for 24 h. Proteins were extracted from the cells for Western blotting. The primary antibodies were as follows: AKT, phosphorylated AKT, ERK 1/2, phosphorylated ERK 1/2, p38 MAPK, phosphorylated p38 MAPK, JNK, phosphorylated JNK (Cell Signaling Technology, Beverly, MA, USA), and β-actin (Santa Cruz Biotechnology, Santa Cruz, CA, USA). The density of each band was quantified with ImageJ software version 1.53e.

### 4.11. Proliferation In Vivo

Hut78 cells were injected subcutaneously into the shaved abdomens of NOD/SCID interleukin-2 receptor γ-chain-deficient (NSG) mice obtained from Charles River Laboratories. On days 0, 4, 7, and 11, the anti-ICOS antibody- or anti-ICOSL antibody-treated groups were intraperitoneally injected with the anti-human ICOS (40 µg/mL) or anti-human ICOSL (40 µg/mL) neutralizing antibodies in 100 µL of PBS. The control group was injected with isotype IgG (R&D systems). The tumor volume was calculated using the following equation: V = π (L1 × L2)/6, where V = volume (mm^3^), L1 = the longest diameter (mm), and L2 = the shortest diameter (mm).

### 4.12. RNA-Seq Analysis

Hut78 cells were co-cultured with anti-ICOS and anti-ICOSL neutralizing antibodies or isotype IgG for 24 h. The total RNA was extracted with Direct-zol RNA Microprep kits (Zymo Research). The cDNA library was prepared and sequenced at Rhelixa Japan to obtain the NGS data (150 bp paired-end reads) using the NovaSeq system (Illumina, San Diego, CA, USA). Read alignment was performed on the mm10 assembly of the mouse genome using STAR (a genome alignment algorithm) [50]. Read normalization, in reads per kilobase of transcript per million, was performed using the HOMER version 4.11 implementation of the R platform [51].

### 4.13. Migration Assay

Hut78 and MyLa cells were treated with the anti-ICOSL antibody. After 24 or 48 h of culture, we assessed migration for 12 h at 37 °C with the human CCL17 protein (0, 50, 100 ng/mL) with 6.5 mm of Transwell with a 8.0 micropore (Corning, Corning, NY, USA).

### 4.14. Statistical Analysis

All statistical data were generated using Prism software version 8 (GraphPad, San Diego, CA, USA). The statistical analysis was performed using the Mann–Whitney *U*-test for comparisons of two groups. Correlation coefficients were determined using the Spearman’s rank correlation test. The Wilcoxon signed-rank test was used to examine the differences between the before and after treatments. Survival analysis was performed using the Kaplan–Meier method, and differences were assessed using the log-rank test. Optical cut-off points were determined by a receiver operating characteristic analysis. *p* values of <0.05 were considered significant.

## Figures and Tables

**Table 1:** ICOS and ICOSL expression in normal skin samples and lesional skin samples from patients with mycosis fungoides (MF) and Sézary syndrome (SS). The intensity of staining in the epidermis was visually quantified as follows: -, no staining; +, slight staining; ++, moderate staining; and +++, strong staining.

		-	+	++	+++
ICOS	Normal	9	0	0	0
	MF (early)	0	2	4	5
	MF (advanced)	0	0	4	11
	SS	0	0	0	5
ICOSL	Normal	9	0	0	0
	MF (early)	0	3	4	4
	MF (advanced)	0	0	6	9
	SS	0	0	0	5

## Data Availability

The data presented in this study are available on request from the corresponding author. The data are not publicly available due to privacy.

## Supplementary Materials

The following supporting information can be downloaded at: https://www.mdpi.com/article/10.3390/ijms27031408/s1.
