# Supplementary material for: Inducible Costimulator and Its Ligand Promote Proliferation and Migration of Tumor Cells in Cutaneous T-Cell Lymphoma
Source: Int J Mol Sci. 2026 Jan 30;27(3):1408. doi: 10.3390/ijms27031408 (PMC12897944; doi:10.3390/ijms27031408)
Supplement: Supplementary file 1 [file ijms-27-01408-s001.zip › TableS1.pdf]

Supplementary Table S1

	0h	6h	12h	24h
p-AKT/AKT	1.0	0.87	0.77	0.67
p-ERK/ERK	1.0	0.97	0.91	0.52
p-p38 MAPK/p38 MAPK	1.0	0.93	0.68	0.53
p-JNK/JNK	1.0	0.97	0.40	0.24
