# Supplementary figures and images for: Inducible Costimulator and Its Ligand Promote Proliferation and Migration of Tumor Cells in Cutaneous T-Cell Lymphoma
Source: Int J Mol Sci. 2026 Jan 30;27(3):1408. doi: 10.3390/ijms27031408 (PMC12897944; doi:10.3390/ijms27031408)

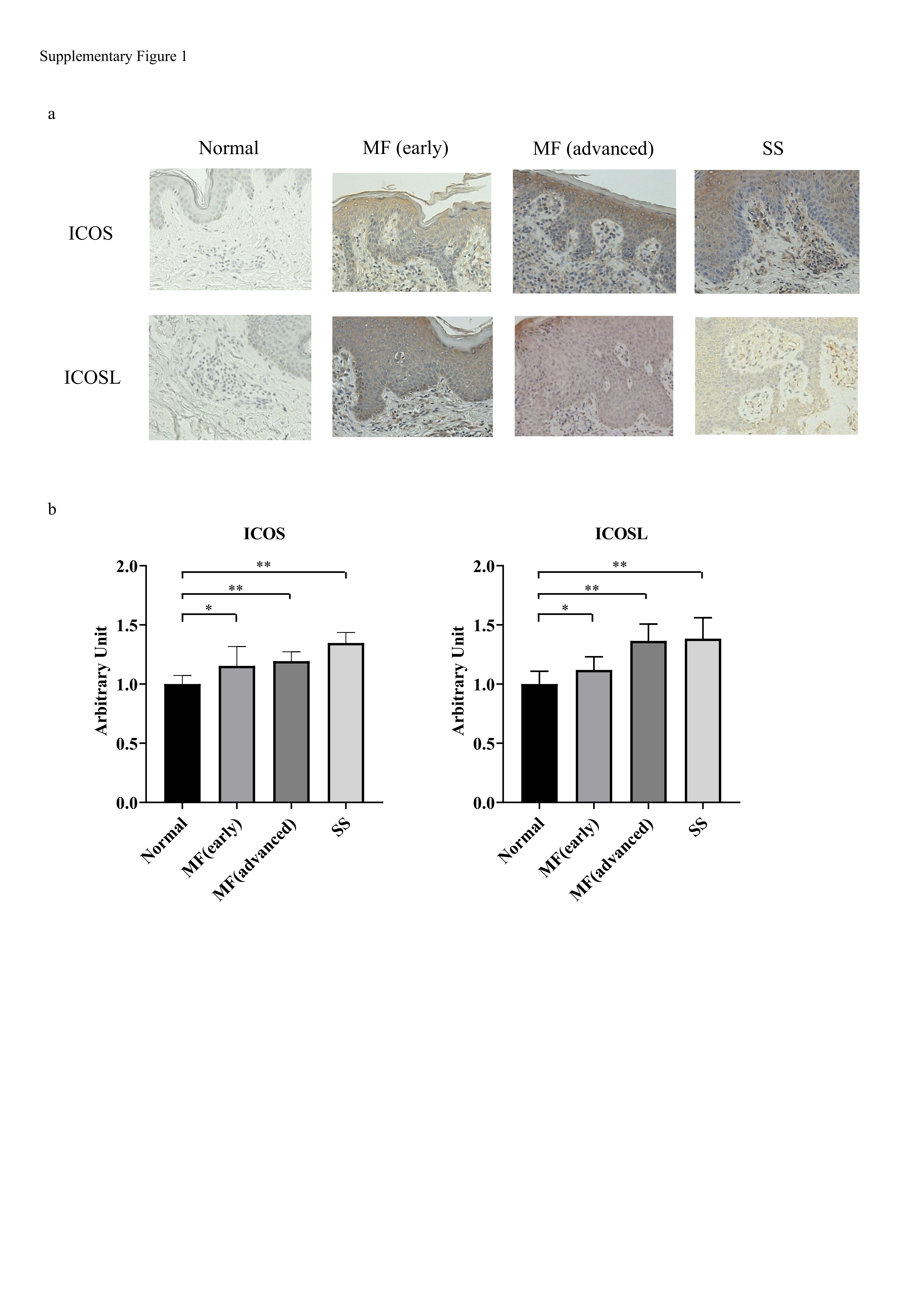

Supplement: Supplementary file 1 [file ijms-27-01408-s001.zip › FigS1.tiff]

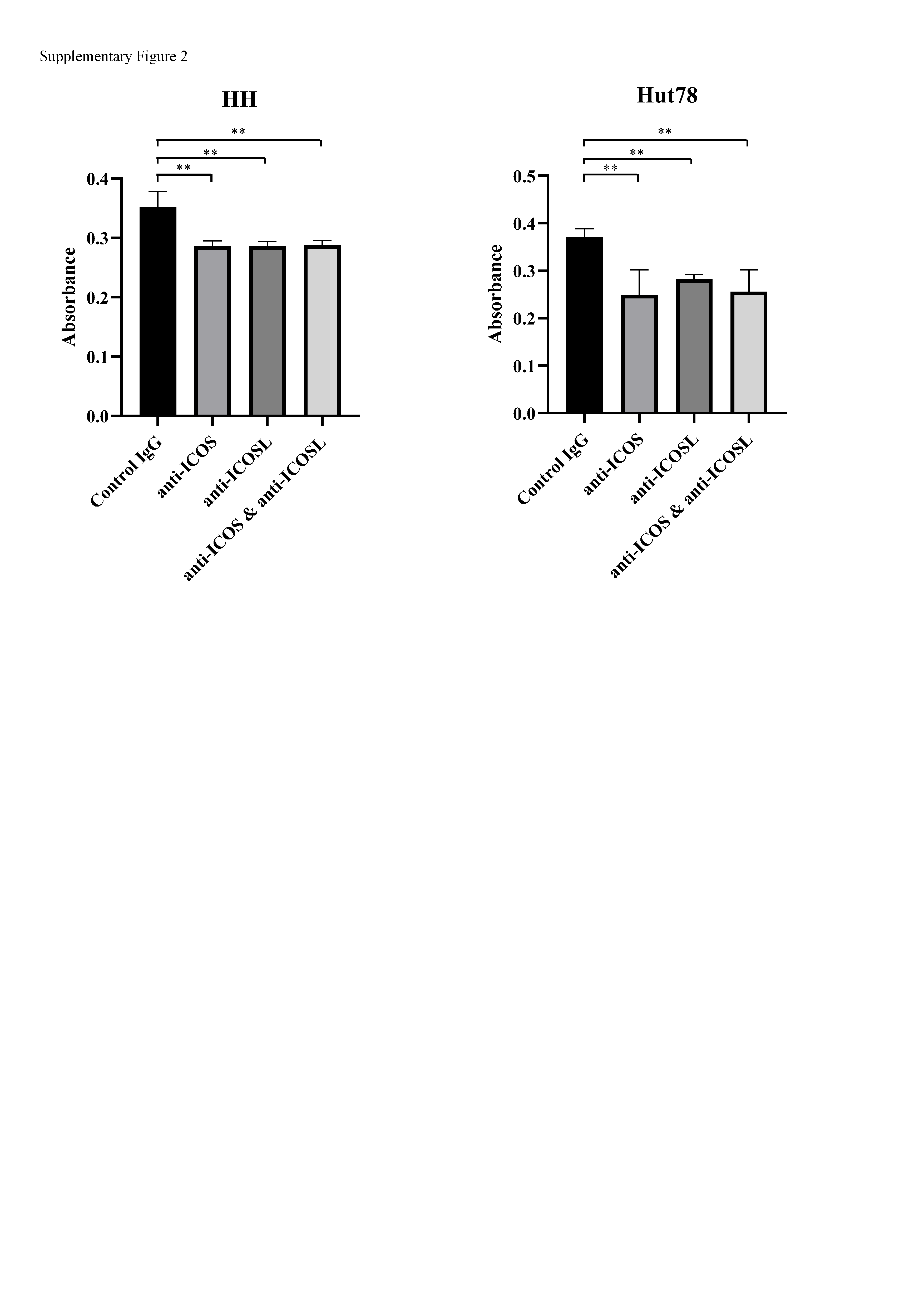

Supplement: Supplementary file 1 [file ijms-27-01408-s001.zip › FigS2.tif]

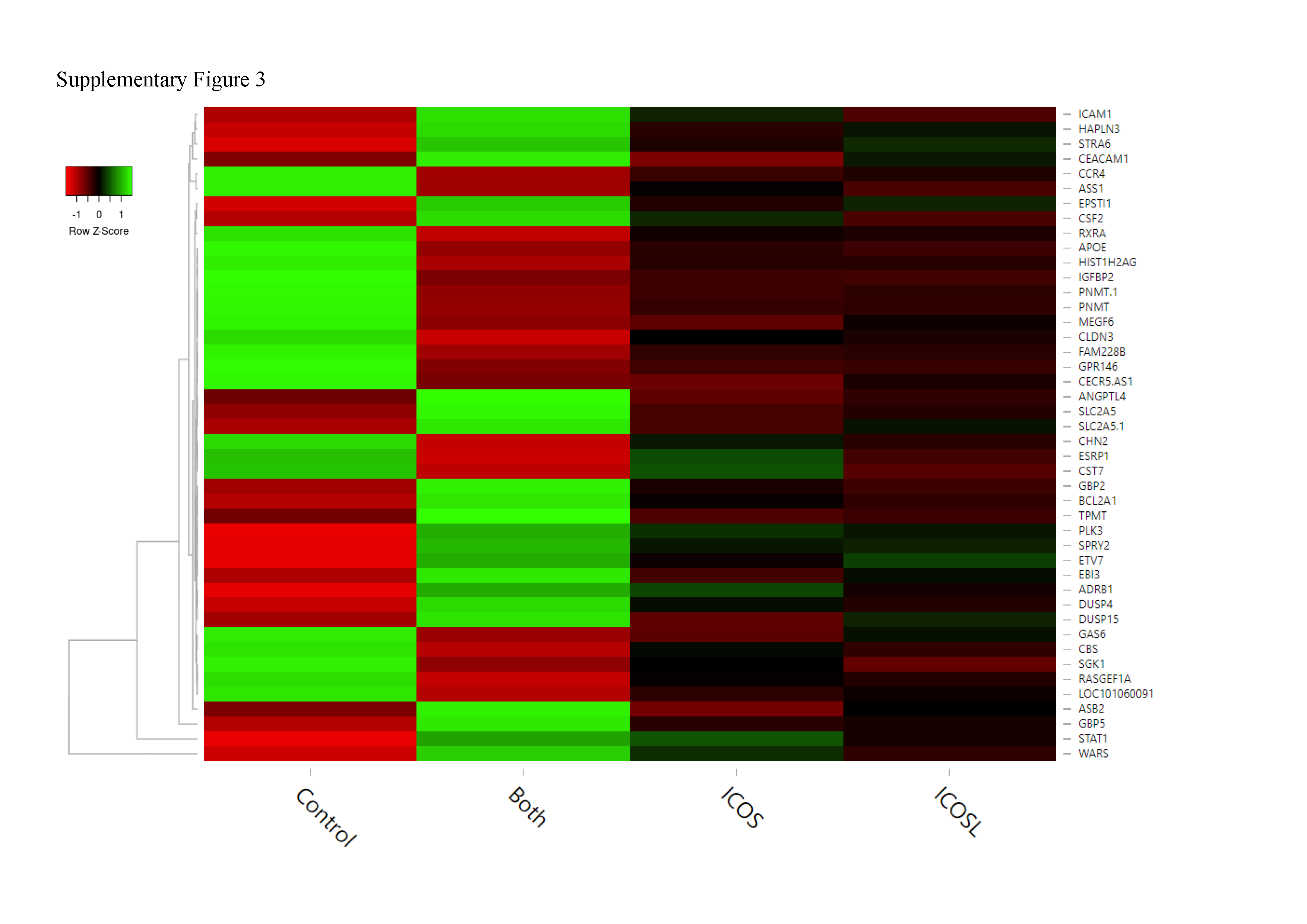

Supplement: Supplementary file 1 [file ijms-27-01408-s001.zip › FigS3.tif]
